# A New Parallel Intelligence Based Light Field Dataset for Depth Refinement and Scene Flow Estimation

**DOI:** 10.3390/s22239483

**Published:** 2022-12-04

**Authors:** Yu Shen, Yuhang Liu, Yonglin Tian, Zhongmin Liu, Feiyue Wang

**Affiliations:** 1 The State Key Laboratory for Management and Control of Complex Systems, Institute of Automation, Chinese Academy of Sciences, Beijing 100190, China; 2 School of Artificial Intelligence, University of Chinese Academy of Sciences, Beijing 100049, China; 3 North Automatic Control Technology Institute, Taiyuan 030006, China; 4 Macao Institute of Systems Engineering, Macau University of Science and Technology, Macao 999078, China; 5 Beijing Engineering Research Center of Intelligent Systems and Technology, Institute of Automation, Chinese Academy of Sciences, Beijing 100190, China

**Keywords:** light field, parallel intelligence, disparity estimation, scene flow, digital twin, virtual real interaction, angular super-resolution

## Abstract

Computer vision tasks, such as motion estimation, depth estimation, object detection, etc., are better suited to light field images with more structural information than traditional 2D monocular images. However, since costly data acquisition instruments are difficult to calibrate, it is always hard to obtain real-world scene light field images. The majority of the datasets for static light field images now available are modest in size and cannot be used in methods such as transformer to fully leverage local and global correlations. Additionally, studies on dynamic situations, such as object tracking and motion estimates based on 4D light field images, have been rare, and we anticipate a superior performance. In this paper, we firstly propose a new static light field dataset that contains up to 50 scenes and takes 8 to 10 perspectives for each scene, with the ground truth including disparities, depths, surface normals, segmentations, and object poses. This dataset is larger scaled compared to current mainstream datasets for depth estimation refinement, and we focus on indoor and some outdoor scenarios. Second, to generate additional optical flow ground truth that indicates 3D motion of objects in addition to the ground truth obtained in static scenes in order to calculate more precise pixel level motion estimation, we released a light field scene flow dataset with dense 3D motion ground truth of pixels, and each scene has 150 frames. Thirdly, by utilizing the DistgDisp and DistgASR, which decouple the angular and spatial domain of the light field, we perform disparity estimation and angular super-resolution to evaluate the performance of our light field dataset. The performance and potential of our dataset in disparity estimation and angular super-resolution have been demonstrated by experimental results.

## 1. Introduction

Progress in computer vision tasks such as object detection [1], semantic segmentation [2], optical flow [3], and disparity estimation [4] has been made in recent decades; however, these tasks mostly rely on monocular or stereo images [5]. The irreversible loss of depth in 2D images is a flaw in nature, and academics have consistently worked to improve algorithms to address this issue. For instance, point cloud [6] characteristics are added to object identification models in autonomous driving to provide a multi-modal framework that makes up for the absence of depth in images. Multiple tasks sharing comparable cues would improve overall performance in a heuristic manner, particularly in edges, according to certain research that combines image disparity estimation and semantic segmentation together [7]. It is obvious that datasets with structural information are crucial because all of the methods mentioned above attempt to employ additional information to generate implicit depth cues.

Recently, some researchers have focused on light fields [8] because, unlike 2D images, light field images implicitly store the directions of rays. Take into account an array light field camera as an example. Cameras with known intrinsic parameters are positioned in a plane or sphere with a specific distance between them. Although views in depth are condensed on the photosensitive plane of the camera, as in conventional cameras, rays projected in different directions from various views record the third dimension, which is perpendicular to the image plane. View images with known intrinsic and extrinsic factors enable more precise reasoning about scene structures. Overall, light field images with richer structural information will perform better as they are more adaptable to current computer vision applications.

However, there are still some difficulties in obtaining a rich and diversified light field datasets. The most notable one is high cost equipment. The current technical framework of light field mainly consists of three types: microlens array [9], camera array [10], and encoded mask [11]. Both of these are too expensive on the market; as an example, the basic edition of raytrix light field [12] costs almost close to $100,000. Such a high price is beyond the acceptance of most researchers and impedes the development of light field technology. An approach is to build a camera array out of inexpensive cameras. These arrays can be placed in a matrix or a circle on the same surface or sphere. Alternatively, you may build a camera array out of inexpensive cameras. These arrays can be set up as a circle or a matrix on the same plane or sphere. Other issues have come up so far, the first of which is the synchronization of many cameras, which is crucial for the perception of dynamic scenes. Another issue is how to arrange the cameras to reduce the redundancy of data from various angles even though images from different views might compensate for one another in dealing with occlusions.

The optimal solution to this issue will significantly improve the perception of autonomous driving’s performance while parameter adjustment is time-consuming and challenging to replicate. Since experiments in the real world are not very efficient, we turn to the virtual world to find a solid plan. To collect static light field scenes with depth and dynamic scene flows, we propose a new light field collection approach based on Parallel Light Fields [13]. The fundamental idea behind Parallel Intelligence is the ACP theory, where *A* stands for Artificial Systems for modeling, *C* is for Computational Experiment for analysis, and then *P* is Parallel Execution for control. Parallel Intelligence, which is characterized by virtual and real interaction, was first proposed by Fei-Yue Wang in 2004 [14] to address management and control of complex systems. Based on the ACP theory, we conduct our experiment in virtual environments, such as sensors’ simulations [15]. To be more precise, we build digital twins of the light, camera, and scenario in the virtual world. By adjusting the parameters of these elements, we can achieve a diverse range of scenarios, which are then captured as light field images by the virtual light field cameras. We can set up numerous deployments till we find an approach that works relatively best to verify the optimum camera arrangement. Our contributions are listed as follows:

1. We present a new large-scale static light field dataset with up to 50 scenes; each scene contains 8–10 different perspectives, covering interior and outdoor scenes, and ground truth is produced, including disparities, depths, surface normals, segmentations, and item postures;
2. Using the free and open-source creation tool Blender [16], we additionally produce a novel light field video with motion ground truth designed for 3D scene flow estimation; in addition to ground truth in static scenes, additional motion information is also collected;
3. With spatial and angular information that have been decoupled, we experiment with disparity estimation and angular super-resolution. Specifically, experimental results demonstrated our dataset’s potential for disparity estimation and angular super-resolution despite the fact that they contain notably higher disparities than the majority of current light field datasets.

## 2. Related Works

### 2.1. ACP Method

Due to its sophisticated concept and domain adaptation, parallel intelligence has recently been used in areas ranging from intelligent transportation, artistic creation, computer vision, and smart sensors. Parallel intelligence was first proposed by Fei-Yue Wang in 2004 to address management and control of complex systems. The ACP theory, which stands for Artificial Systems for modeling, Computational Experiments for analysis, and Parallel Executions for control, is the foundation of parallel intelligence. Shen [17] consulted the ACP theory and proposed the Parallel Light Fields concept in an effort to find a solution because the acquisition and control of rays are extremely complex and have significant meanings. The proposed Parallel Light Fields framework divides light fields into three parts: digital twins of the light sources, virtual cameras, and light fields, which together constitute artificial systems. The effectiveness of these experiments was then evaluated by constructing several scenarios with various illumination by adjusting the intensity and direction of the light sources; a typical application is shadow detection and removal for illumination consistency by tuning directions of lights [18].

As we know, light field images implicitly record 3D structural information within multiple views; however, these viewpoints have a lot in common and cause data redundancy, which may impede the efficiency of algorithms; therefore, how to arrange light field camera arrays becomes a significant issue which has never been addressed. In the virtual and real interactive Parallel Light Fields framework, a lot of hypotheses can be verified that are difficult to conduct in the real world. On the other hand, to perceive indoor and outdoor scenes, different scale objects in the environment have different demands on light field cameras. For example, if you want to estimate depth and perform semantic segmentation, scales of the scene may be within a few meters, and light field camera arrays with small baseline distance may be able to meet the demand. However, in autonomous driving, cameras must be able to see tens to hundreds of meters in front of them. For this purpose, larger baseline distances are considered necessary, and the optic axes of various cameras must be adjustable from parallel to intersect such that light field cameras can adapt to a strong afternoon sun [19]. The aforementioned experiments necessitate a significant amount of manpower; however, we can leverage parallel systems to do a variety of computational experiments and verify them in real systems. The convergence of the whole closed system to the optimal states may be prescripted by interactions between virtual and real systems.

### 2.2. Light Field Data Generation

The preparation of a dataset is the preliminary step in conducting research on light fields. Common light field datasets include HCI old, HCI new [20], 4DLFVD [21], INRIA lytro [22], Stanford Gantry [23], as well as others. They can be categorized into three groups based on how they were acquired: utilizing commercial light field devices, manually building camera arrays, and using software rendering. Some of these datasets lacking the ground truth are therefore difficult to utilize for accurate benchmarking. As a passive perception technique, INRIA lytro creates a light field dataset using the commercial personal lytro light field cameras. However, due to perspective limitations, only partial views of the scenes may be recorded. Additionally, since the fundamental characteristics of the light field cameras have already been calibrated, it is hard to modify them for task-specific scenarios. A high-density 4D light field video dataset called 4DLFVD was compiled by camera matrices. They construct collection systems made up of 10×10 1080p monocular video cameras distributed uniformly in the same plane and direction and with a resolution of 1920×1056 pixels. Their dataset consists of nine groups of videos with both interior and outdoor situations. The HCI dataset was virtually created using the free and open-source software Blender. It is comprised of 20 photorealistic light field scenes with a resolution of 512×512 and 4 stratified scenes.

A recent study has attempted to determine the association between various angulars and then augment the light field dataset. This is in line with the development of deep learning. An important concept is angular super resolution. To increase the effectiveness of processing light field data, Yu [24] designed a series of disentangling convolutions to split up high-dimensional light field images into numerous low-dimensional subspaces. By combining spatial and angular features, they unsampled 2×2 light field viewpoints to denser 7×7.

Others include the increasingly popular Neural rendering technique known as NeRF [25], which outputs a volume density and view-dependent emitted radiance at a given spatial position using a 5D coordinate system (spatial location (*x*,*y*,*z*) and viewing direction (θ,ϕ)) as input. Suhail [26] presented a model that may simulate non-Lambertian effects as well as view-dependent impacts of synthesized perspectives. In order to produce the color of a target ray, they devised a two-stage transformer-based approach that first aggregates features along epiploar lines and then aggregates features along reference views.

### 2.3. Light Field Disparity Estimation

According to epipolar geometry, disparity [27] refers to the displacement of the same pixel in neighboring views. Since depth and disparity have an inverse proportional relationship, calculating disparities allows us to determine the scene’s depth. This is one of the main applications for light fields. The widely used stereo cameras are based on this principle, and autonomous driving is a typical implementation. Due to the fact that discrepancies between pixels are determined by locating relevant pixels, stereo matching issues can only occur when cameras are placed on a rig of the same height. If occlusion then arises, the problem will become more severe. However, this can be mitigated by employing light field cameras, as in light field formulation, we can additionally acquire angular dimension, which provides implicit indications of scene structure. For the purpose of estimating the disparity of light fields, Tsai [28] presented an attention-based view selection network. They created a view selection module that produces an attention map that indicates how important each view is.

### 2.4. Light Field Angular Super-Resolution

Reconstructing light field images from a lower angular resolution to a higher one is the goal of light field angular super-resolution [29]. Since we are aware that various viewpoints in a light field image correlate to various disparities, angular super-resolution is always combined with depth cues and used as an auxiliary task. One technique that makes use of depth is to implicitly concatenate depth features with extracted angular and spatial features in different phases and then refine the network to produce upsampled light field images [30]. Another technique is warping disparities with the reference view and blend to the target view [31]. To eliminate trade-offs between the spatial and angular domains and fully utilize the abundant light field data, Cao [32] proposed a multi-model fusion in light field angular super-resolution estimation. By effectively exploiting the intrinsic geometry information, Jin [29] developed an end-to-end learning-based approach capable of angularly super-resolving a sparsely sampled light field with a large baseline.

## 3. Approaches

**Figure 1:**
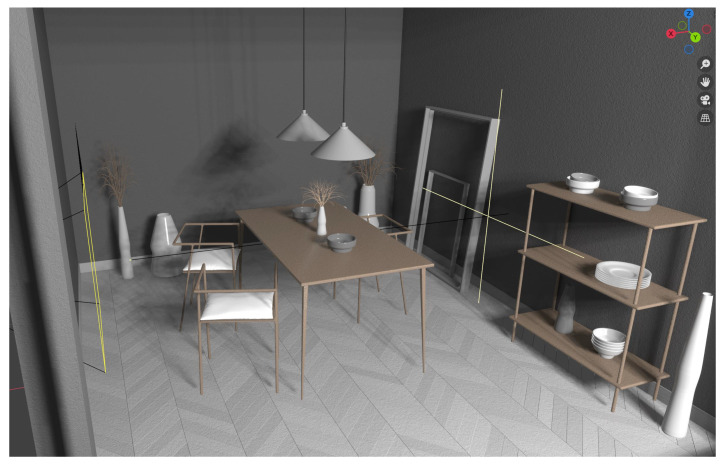
The Blender interface used to generate light field images.

The first step in Parallel Light Fields is the construction of Artificial Systems, on top of the Computational Experiments and Parallel Executions that featured as virtual and real interaction. In this study, we use a virtual platform called Blender, an open source creation tool, to carry out light field research. First of all, in the virtual world, we can create a variety of scenarios tailored for specific tasks such as autonomous driving recognition, indoor scene perception, factory assembly line for industrial inspection, and so on. An example indoor scene is as shown in Figure 1; as we can see, the virtual scenes with naturalism desks, chairs, bowls, ceiling lamp, and others with concrete 3D sizes created within the Blender virtual environment are observed from the perspective of a camera. Another view camera is marked in yellow from the left side of the scene. With full scene known parameters, we can flexibly arrange objects and lights to construct scenes as we need. Afterwards, since Blender offers a Python API, it is convenient for us to make user-defined add-ons, as we need to self-define virtual light field camera arrays used to collect light field images for subsequent tasks. In order to accomplish our objectives, we developed a light field add-on that simulates the sampling of light field cameras in real life. To make it, we adjusted the light field camera array layout to make the baseline distances between cameras adjustable. To be more precise, a larger baseline is more appropriate for distant and expansive circumstances, and vice versa; this is in line with how our eyes see. On the other hand, we can explore reducing redundancy of overlapped camera views by tuning baseline distances among cameras as a distribution as uniform or exponential. The basic light field camera parameters we can adjust in Blender are shown as follows in Table 1:

The other two associated elements are digital twin light sources and digital twin scenarios. Depending on whether a scene is indoors or outside, distinct kinds of light sources are available, such as spot, dot, area, and sun light. Light field cameras may implicitly record ray direction; therefore, in addition to color rendering, we can conduct research by taking ray direction into account. For instance, in autonomous driving, afternoon sunshine shining straight into the camera lens can cause potential risks. Humans can switch to a different view to avoid this situation, but an autonomous vehicle cannot. A parallel system is an excellent setting for these tests. To solve this problem, we consult Parallel Light Fields, and one possible solution we come up with is spherical light field camera arrays by positioning cameras on surfaces with variable radii. With the help of some computer vision or graphical algorithms, we can reason about the variable directions of lights captured by different camera lenses in different positions and filter noisy lights to obtain final clear light field images.

### 3.1. ACP Based Optimal Camera Array Deployment

Stereo images, as we are aware, will have better depth estimation accuracy than monocular images due to the additional 3D structure information provided by a second view. For the same reason that we use light field images, more viewpoints of the image will provide significantly more cues for depth inference. Even if we have up to 9×9 angular resolution, not every view has an equal effect on the final depth estimation. Adding more views therefore increases data volume and the computational burden. Additionally, these additional view images have a high level of redundancy. As far as we are aware, there is no task that specializes in optimal view selection and can extract the most information from light field images with the smallest number of views. For tasks like depth estimates, some early studies randomly selected a portion of the viewpoint. It becomes difficult and necessary to figure out how to choose the most appropriate viewpoints in order to increase efficiency. We discovered that the optimal camera array deployment may be accomplished by adjusting the distances between nearby cameras and the camera sphere in order to strike a compromise between scene depth accuracy and view redundancy. The solution to this issue will have a substantial impact on the creation and design of light field cameras in practical applications. It would be preferable if we could intelligently adjust the baselines to make our light field cameras task-specific.

The maximum recovery of 3D structural information of small objects and large-scale scenes have differing demands on camera array configurations, making the optimal view selection significant in light field camera design as well. Take camera arrays as an example. For instance, camera arrays can be placed in a sphere or a plane, and the distance between neighboring cameras can either be equal, linearly growing, inverse proportional, or exponential. It is unrealistic to conduct experiments on every single mode to figure out the optimal deployment for every single case in the real world. Due to the light field camera’s ability to restore implicit 3D structures, we believe it may eventually replace conventional cameras. Making the light field camera more adjustable will be a significant and fascinating challenge. The inflexibility may have been one of the challenges to the development and popularity of commercial light field camera devices.

**Figure 2:**
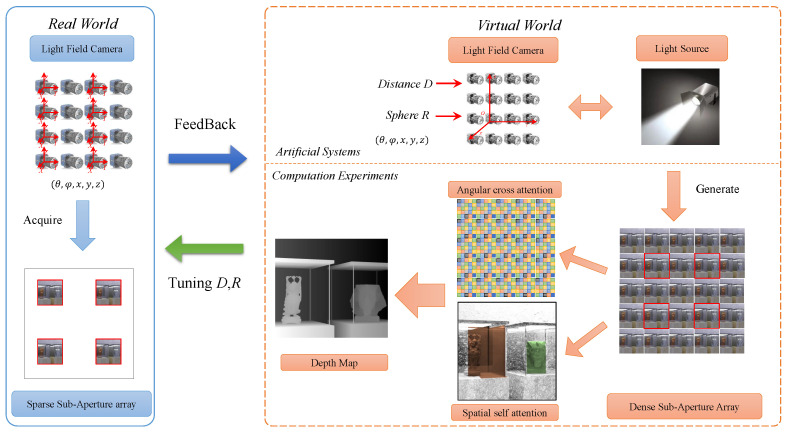
Framework of ACP based light field generation.

We propose a solution in this essay that is based on the ACP theory as shown in Figure 2. The Parallel Intelligent aspect of the ACP theory is abstracted to include both virtual and real interaction. Create artificial systems that are similar to real systems before conducting computational experiments there. Small data in the virtual world may then be expanded to big data for intelligent algorithms, and once smart knowledge has been obtained, it can be used in real systems for validation. Iteratively repeating this process until a complete closed loop is achieved, the systems work in an interactive way between the real and virtual worlds until final convergence. Feedback from actual systems will direct the virtual systems to update settings iteratively.

To be specific, the Blender suite is very flexible and a Python API is accessible, which means we can compile our own light field add-ons. In stereo matching, the depth and disparity follow the rule as

(1) $$depth=\frac{fb}{disparity},$$

where *b* is baseline between adjacent cameras, *f* is the focal length, and, if disparity is fixed, depth is proportional to baseline distance; this is the same with our eye.

Therefore, in our settings, we choose to focus on closer scenes with cameras that are closer to the central view and faraway scenes with cameras that are farther from the central view. By adjusting camera array baseline distances to achieve a full focus mode, we carry out computational experiments in the virtual space. The scene images are then recorded using rendered light field cameras.

### 3.2. Light Field Static Scene Generation

By utilizing the free and open source Blender 3D creation platform, we have published a new light field image collection in this work. A virtual light field camera in the form of camera arrays was used to take the scene images with diverse lighting conditions such as sunshine, spot light, and dot light. To build the scene in Blender, we employed a number of 3D models, including indoor rooms, outdoor houses, and streets.

The 3D model that is used to build the scene in our settings comes from two sources. First, it may be acquired from a website that provides free 3D models; these models were all generated using the Blender software. Even though some of them are not very realistic, they are nevertheless useful because we are aware of all the 3D measurements and may modify the model to our preference. Second, in order to lessen the impact of natural sunlight on the rendered scene, we also collected the 3D models of a few additional objects utilizing scanning devices like RGBD cameras and iPADS. These additional objects, which primarily include real-world cars, were collected on cloudy days rather than sunny ones. In this manner, we can produce a variety of scenes in batches to meet the demands of our tasks by completely utilizing the large real-world and virtual 3D models. We use the cycle engine to imitate natural qualities of light and increase rendering realism at the expense of rendering time. The depth is generated using Blender’s Z pass, where the Z pass values correspond to the length of the ray from the object’s surface point to the camera’s pinhole. In contrast to the Eevee engine, the physical Cycle engine can track rays and support a variety of complicated materials, producing in more realistic results. Light field images require containing rays’ directions in addition to their appearance and intensity, in contrast to conventional 2D images, hence the cycle engine is the ideal option. The notion behind the volume rendering used in the well-known Neural Rendering network is comparable to how information along the ray is recorded in the cycle engine. We reduce the sampling rate, and the maximum light path bounces to a relatively smaller amount with little loss in rendering quality in order to speed up rendering.

**Figure 3:**
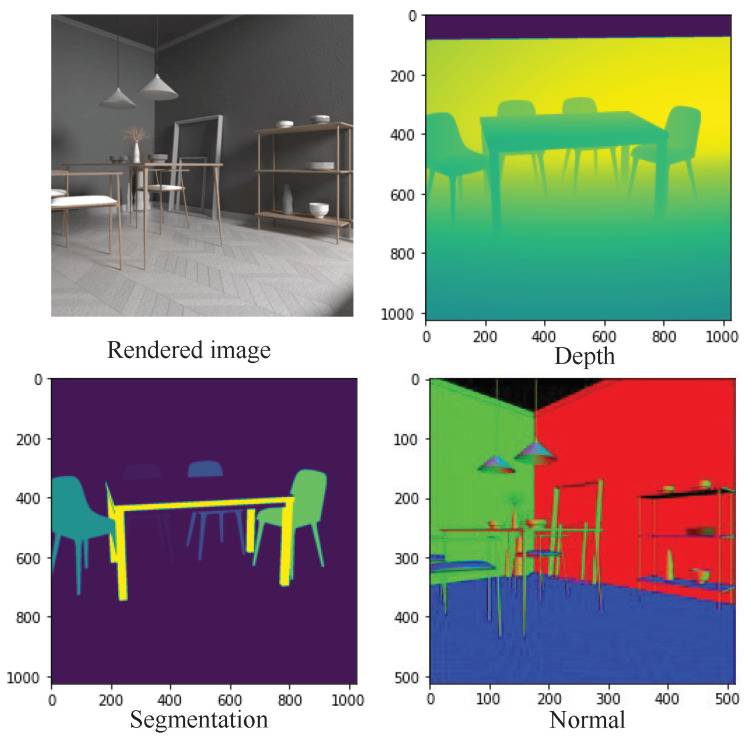
Our rendered light field image depth, segmentation and normal groundtruth maps.

Our light field image has a spatial resolution of 1024×1024 and angular resolution up to 9×9. To verify how baseline distance between adjacent cameras will affect the depth estimation performance, we set two versions of angular resolution with 7×7 and 9×9 spatial resolution and the lower the spatial resolution, the longer the baseline distance. In the popular HCI dataset, the training dataset includes 16 simple scenes, and only the depth/disparity ground truth is available; on the contrary, with the HCI dataset, as depicted in Figure 3 our captured scene is more complex, and provides ground truth including the depth/disparity for depth estimation, a normal that indicates the ray direction, and semantic cues for segmentation. In addition, the HCI dataset focuses on a relatively simple scene and distances between camera lenses are relatively small; their disparities are within –4 to 4 pixels, our goal is trying to make a dataset that is suitable for large scale scenarios, so our light field dataset has disparities ranging from 0 to 200 pixels, which is similar to current 2D autonomous driving datasets like KITTI. We hope that our light field dataset is capable of training current perception algorithms by exploiting 3D structural features more effectively and boosting their performance in tasks like depth estimation and object detection.

**Figure 4:**
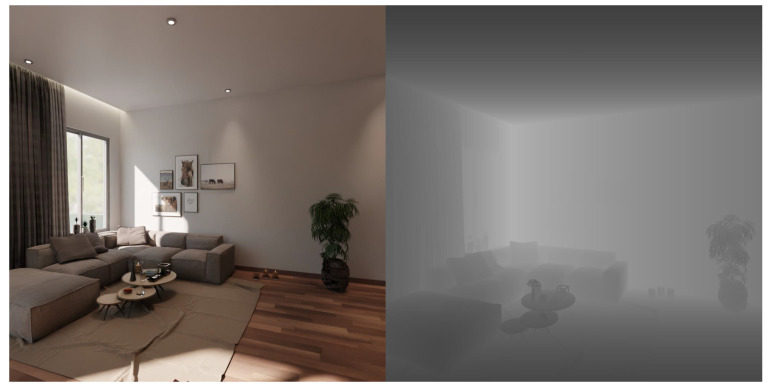
Our light field image and depth ground truth rendered by Blender.

Our light field image has an angular resolution of up to 9×9 and a spatial resolution of 1024×1024. We set up two versions of angular resolution with 7×7 and 9×9 spatial resolution, and the lower the spatial resolution, the greater the baseline distance, to investigate how baseline distance between neighboring cameras will affect the depth estimation performance. The popular HCI dataset only has depth/disparity ground truth available for 16 simple scenes in the training dataset. In contrast, our captured scene has depth/disparity for depth estimation, a normal that shows the ray direction, and semantic cues for segmentation. In addition, the HCI dataset focuses on relatively simple scenes and camera lens distances are relatively small; their disparities are within −4 to 4 pixels. Our goal is to create a dataset that is appropriate for large scale scenarios, so our light field dataset has disparities ranging from 0 to 200 pixels, as presented in Figure 4, which is similar to current 2D autonomous driving datasets like KITTI. We anticipate that, by more efficiently exploiting 3D structural features, our light field dataset will be able to train current perception algorithms and improve their performance in tasks like depth estimation and object detection.

### 3.3. Light Field Dynamic Scene Generation

Videos are merely 2D frame sets with depth discarded, making them unsuitable for 3D motion estimates. In order to examine dynamic characteristics of the scene, we require 3D models with time-varying motions. We must therefore create dynamic light field videos for studying scene flow, as we do with static scenes. Comparing dynamic scenes to static scenes, merely making every object move over time is insufficient for the creation of a dynamic scene; instead, the objects in the scene must be connected by correlation relations for the scene to have any semantic significance. These semantic signals will be useful in tasks like object tracking based on light field scene flow prediction. For instance, a man should run on a road instead of on the river, as well as a boat should float on the river instead of fly in the air.

**Figure 5:**
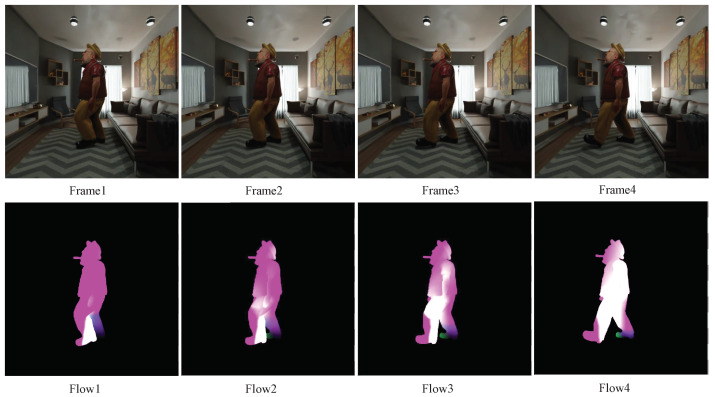
Rendered images and corresponding flows.

Thanks to the availability of large-scale, free 3D animated models in full 3D sizes, we are now able to create dynamic sceneries in the form of optical flow. We can render a whole scene animation and capture a dynamic full scene using our light field camera after importing 3D dynamic human models into our static scenes. In Blender, the vector pass in the data module can generate relative pixel displacement between adjacent frames, which can subsequently be used to acquire optical flow. The generated flow dataset is shown in Figure 5. The upper row depicts a dynamic scene rendered with a moving person in a static environment, and the lower row depicts comparable flows of the dynamic scene. The colors in the flow images denote the directions and magnitudes for speeds, respectively. Our dynamic scene light field images have the same spatial and angular resolution as our static scene light field images, and each scene’s whole 6D light field dataset spans 150×9×9×1024×1024×3 dimensions, with 150 being the frame. For the purpose of future light field image-based perception, we also obtained ground truth of segmentations, depths, normals, and object poses in addition to flows.

## 4. Experiments

### 4.1. Light Field Disparity Estimation

Our light field dataset is more suitable to transfer to various 2D image-based deep learning algorithms since it is indoor and outdoor focused, with large depth compared to many other public datasets. We trained the widely used light field disparity estimation network DistgDisp using our light field dataset and the HCI light dataset, respectively, to validate our proposed dataset in depth estimation. In the DistgDisp [24] benchmark, the subaperture input images are first rearranged into a macro pixel image, and fed to eight spatial residual blocks for spatial information incorporation; afterwards, a series of disparity-selective angular feature extractors were introduced to disentangle the disparity information from the macro pixel image. Similar to conventional stereo matching, the extracted features are merged to generate a cost volume. The aggregated cost is then used to regress the final disparity using 3D convolutions. Although the network used in this method is not particularly complex, which is simply a fully convolutional extractor without an attention module, it achieves a mean square error (MSE) value of 0.01896 and Badpix0.01 values of 23.328 on the HCI light field dataset, and, as of the time they submitted their results, it was ranked second:

(2) $$MSE=\frac{1}{H*W}\sum_{\begin{array}{c} i \end{array}}^{H}\sum_{\begin{array}{c} j \end{array}}^{W}{\left(disp_{esti}\left(i,j\right)-disp_{gt}\left(i,j\right)\right)}^{2}$$

The smaller disparity of the HCI dataset, on the one hand, is thought to be responsible for their strong performance. The HCI dataset has a maximum disparity of 4 pixels, which indicates that the 9×9 view photos have a lot in common and that redundant information improves network efficiency. However, compared to monocular or stereo pictures, the combination of spatial, angular, and epipolar properties offers significantly more structural information and contributes to enhancing the disparity results.

Since our dataset has much greater disparity than the HCI dataset, we modified the refocus augmentation to adapt to our disparity range in the data augmentation stage, and the initial learning rate and decay are recalculated as $1\times 10^{-4}$ and 0.75, respectively. As a result, we use DistgDisp as our baseline to evaluate our light field dataset. We have not masked out transparent or reflective parts, in contrast to the training on the HCI dataset, even though they might impair the performance of disparity estimation. Finally, we reach an MSE of 2.352, which is 120 times greater than on the HCI dataset. Given that the maximum disparity on our dataset is up to 200 pixels, we believe this is a reasonable threshold.

**Figure 6:**
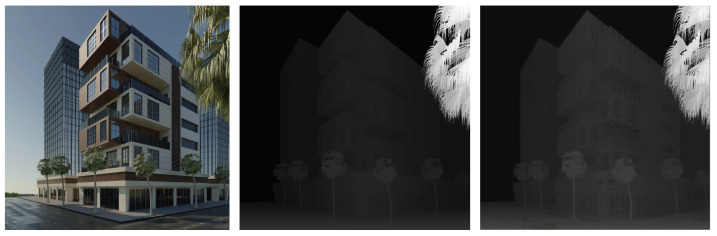
From left to right are rendered images, disparity ground truth, and estimated disparity.

As can be seen, the estimated disparity map in Figure 6 can recover certain structural elements beyond the captured depth ground truth.

On the other hand, we also conduct experiments on the epipolar plane images based light field disparity estimation method named EpiNet [33] on our dataset. We make some modifications: the initial learning rate and decay are recalculated as $5\times 10^{-6}$ and 0.5, respectively; different from that trained on the HCI dataset, we do not mask out any reflection region pixel but train the whole area. We finally adopt 40 scenes and each with eight perspective light field images for training, the results are shown in Table 2 the mean square error (MSE) is used as a metric, and our result is 1.245. Since our dataset has a disparity of up to more than 100 pixels and is larger than [−4,4] in the HCI dataset, in addition to the baseline distance also being larger and there being no other light field dataset similar to ours, it is hard to make a relatively fair comparison.

### 4.2. Light Field Angular Super-Resolution

The angular super-resolution experiment, often known as viewpoint synthesis or reconstruction, is another fundamental light field experiment. In order to evaluate on our dataset, we additionally use the DistgASR disentangling strategy. To take advantage of performance in scenes with large disparities, we conduct light field angular super-resolution. To effectively exploit correlations within each domain, DisgtASR [24] also introduces two additional horizontal and vertical epipolar plane images (EPIs) feature extractors in addition to the spatial feature extractor and angular feature extractor. The angular feature extractor takes in an angular resolution of 3×3 and dilation by 1 to calculate correlations between different views, and the spatial feature extractor with a kernel by 3×3 and dilation by angular resolution, respectively, to extract features within each subaperture image as conventional convolutions. Features extracted from these four modules are concatenated to input to two spatial feature extract modules, and the aforementioned modules together composite the Disentangle Block (Distg-Block). After a few Distg-Blocks, a 1×1 convolution aggregate features in to dimensions of $1\times 1\times \beta^{2}A^{2}C$ where β is the upsampling rate, *A* is original angular resolution, and *C* is the feature channels. A pixel shuffle module was finally used to obtain the final upsampled result with a size of βA×βA×C; here, we set β as $\frac{7}{2}$ and *A* is 2. We follow the training strategy in DistgASR and use the Peak Signal Noise Ratio (PSNR) and Structural Similarity (SSIM) as the metric to evaluate our result.

**Figure 7:**
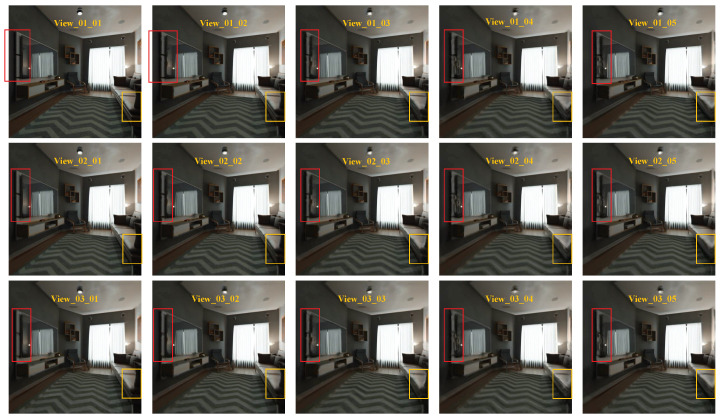
Results of light fields angular super-resolution on our dataset.

**Figure 8:**
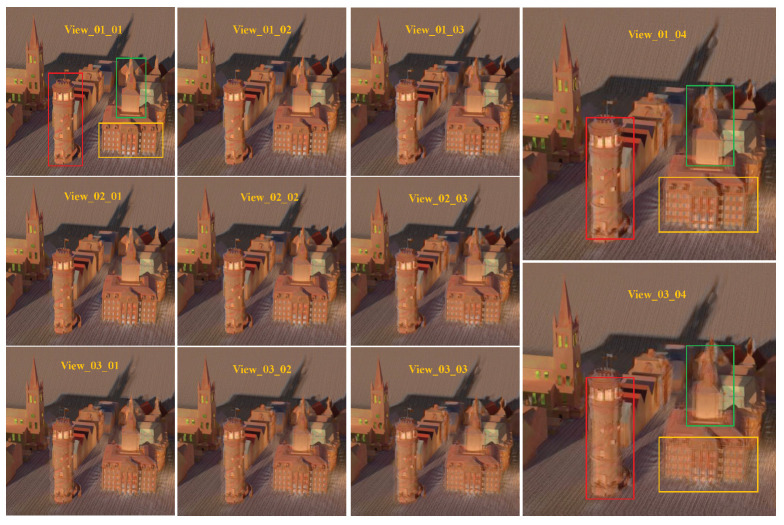
Results of light fields angular super-resolution on HCI dataset with high depth.

**Figure 9:**
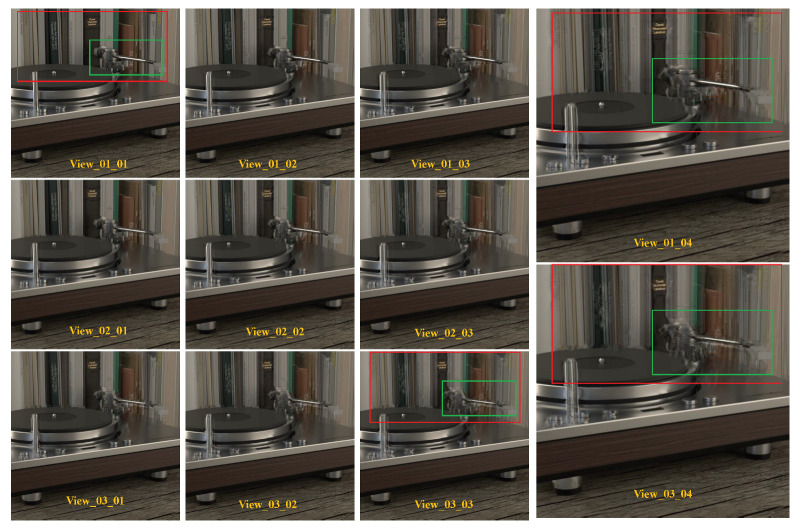
Results of light fields angular super-resolution on HCI dataset with low depth.

To reconstruct the other 45 views, we choose the four corner views from the 7×7 views as our input. We achieve a PSNR and SSIM of 30.135487 and 0.942329 in the 2×2 to 7×7 angular super-resolution task show in Table 3. On the contrary, the DistgASR trained on the HCI dataset achieved scores of PSNR 34.7 and SSIM 0.974, which are better than in our dataset. We attribute this to our larger scale disparity, which results in significant displacement of pixels in adjacent views. As illustrated in Figure 7, we chose the upper left 15 views for demonstration, with View_01_01 acting as the input and the remaining views being the reconstructed views. To illustrate our finding that the reconstruction perspective becomes blurrier the farther away it is from the input perspective, we depict the same spatial location in red and yellow boxes drawn from several perspectives. As we can see, the mirror and its edge are becoming blurry in the red boxes from upper left to lower right, and in the yellow boxes as well. We believe this is because of the low roughness and shallow depth. We also trained DistgASR on the HCI dataset with two disparity ranges for verification and depict the bigger disparity ranging from −3.6 to 3.5 in Figure 8 and the smaller disparity ranging from −1.6 to 1.2 in Figure 9. The blurred areas in larger disparity inputs frequently occur in the lower depth region. We believe that this is because the lower depth corresponds to larger disparities, which means there is greater movement between adjacent view images and may result in errors. In contrast, in small disparity areas, the network is better capable of reconstructing the scene. In conclusion, we think that larger disparity may be the reason for performance degradation in angular super-resolution, since disparities in our dataset are much larger than in the HCI dataset; even though the PSNR and SSIM in our dataset are lower than in the HCI dataset, we think our performance is better. In addition, since no dataset currently exists with a relative disparity range as wide as ours, we expect that using our dataset would significantly improve the performance of existing algorithms developed using monocular or biocular datasets and expand their application to autonomous driving perception scenarios.

(3) $$PSNR=10\log_{10}\left(\frac{{\left(2^{n}-1\right)}^{2}}{MSE}\right)$$

(4) $$MSE=\frac{1}{H*W}\sum_{\begin{array}{c} i \end{array}}^{H}\sum_{\begin{array}{c} j \end{array}}^{W}{\left(Ref_{img}\left(i,j\right)-Rec_{img}\left(i,j\right)\right)}^{2}$$

Light fields’ angular super-resolution is the inverse of light fields capturing, and the PSNR and SSIM can be seen as an indicator of correlation between the reference view image and the reconstructed view image. Based on these observations, we can choose views with the least redundancy by adjusting the baseline distances; to be concrete, if the recovered views are blurred, the PSNR and SSIM are low; then, we think a camera lens should be deployed in that position to provide some complementary cues. We will conduct such research in the future work.

## 5. Conclusions

In this paper, we produce a brand-new synthetic light field dataset specifically designed for static scene depth estimation and dynamic scene flow estimation by compiling an add-on using the free and open-source Python API of the Blender suite. Our static light field scene images include both indoor and outdoor scenarios. When compared to other light field datasets, our dataset is larger in terms of scene scale and depth, and it explores the potential of light field images for indoor 3D reconstruction. With the help of the Python API, light field research may be applied to the context of autonomous driving, which will, in our opinion, significantly enhance the effectiveness of visual perception algorithms. Additionally, since it is challenging to collect ground truth for 3D dynamic models, we also create a dynamic 3D motion dataset for exploring scene flow. Thanks to Blender’s virtual environment, where sizes and motion parameters are known, ground truth is also much easier to collect.

We also evaluate main stream disparity estimation and angular super-resolution algorithms on our static scene dataset, with little modification on the training strategy of the network, even though results trained on our dataset are not as good as those trained on the HCI dataset, we think it is because our dataset is more complex and has a larger disparity which is more suitable for algorithms designed for current autonomous scenarios, and our future work will fully exploit large scale scenes.

## Figures and Tables

**Table 1:** Camera parameters set in the Blender platform.

Focal Length	Baseline Distance	Sensor Size	Image Size	Angular Resolution
39 mm	0.125 m	36 mm	1024×1024	9×9

**Table 2:** Disparity estimation results comparision between HCI dataset and uur dataset.

Model	HCI (MSE)	Our Data (MSE)
DistgDisp	0.01896	2.352
EpiNet	0.01280	1.245

**Table 3:** Results comparision between HCI dataset and our dataset.

Dataset	PSNR	SSIM
HCI	34.7	0.974
Our data	30.135487	0.942329

## Data Availability

Not applicable.

## Abbreviations

The following abbreviations are used in this manuscript:

SSIM	Structural Similarity
PSNR	Peak Signal Noise Ratio
API	Application Interface
ACP	Artificial systems, Computational Experiments, Parallel Execution
EPI	Epipolar Image
